# Physicochemical characterization, cytotoxic effect and toxicity evaluation of nanostructured lipid carrier loaded with eucalyptol

**DOI:** 10.1186/s12906-021-03422-y

**Published:** 2021-10-07

**Authors:** Mira Nadiah Mohd Izham, Yazmin Hussin, Nurul Fattin Che Rahim, Muhammad Nazirul Mubin Aziz, Swee Keong Yeap, Heshu Sulaiman Rahman, Mas Jaffri Masarudin, Nurul Elyani Mohamad, Rasedee Abdullah, Noorjahan Banu Alitheen

**Affiliations:** 1 Department of Cell and Molecular Biology, Faculty of Biotechnology and Biomolecular Sciences, Universiti Putra Malaysia, 43400 UPM Serdang, Selangor Malaysia; 2 China-ASEAN College of Marine Sciences, Xiamen University Malaysia, 43900 Sepang, Malaysia; 3 Department of Physiology, College of Medicine, University of Sulaimani, Sulaymaniyah, 0046 Republic of Iraq; 4 UPM-MAKNA Cancer Research Laboratory, Institute of Bioscience, Universiti Putra Malaysia, 43400 Serdang, Selangor Malaysia; 5 Biotechnology Research Institute, Universiti Malaysia Sabah, 88400 Kota Kinabalu, Sabah Malaysia; 6 Faculty of Veterinary Medicine, Universiti Putra Malaysia, 43400 UPM Serdang, Selangor Malaysia

**Keywords:** Eucalyptol, Loaded nanocarrier, Sub-chronic toxicity assay, Anti-breast cancer efficacy, Promising outcome

## Abstract

**Background:**

Eucalyptol is an active compound of eucalyptus essential oil and was reported to have many medical attributes including cytotoxic effect on breast cancer cells. However, it has low solubility in aqueous solutions which limits its bioavailability and cytotoxic efficiency. In this study, nanostructured lipid carrier loaded with eucalyptol (NLC-Eu) was formulated and characterized and the cytotoxic effect of NLC-Eu towards breast cancer cell lines was determined. In addition, its toxicity in animal model, BALB/c mice was also incorporated into this study to validate the safety of NLC-Eu.

**Methods:**

Eucalyptol, a monoterpene oxide active, was used to formulate the NLC-Eu by using high pressure homogenization technique. The physicochemical characterization of NLC-Eu was performed to assess its morphology, particle size, polydispersity index, and zeta potential. The in vitro cytotoxic effects of this encapsulated eucalyptol on human (MDA MB-231) and murine (4 T1) breast cancer cell lines were determined using the MTT assay. Additionally, acridine orange/propidium iodide assay was conducted on the NLC-Eu treated MDA MB-231 cells. The in vivo sub-chronic toxicity of the prepared NLC-Eu was investigated using an in vivo BALB/c mice model.

**Results:**

As a result, the light, translucent, milky-colored NLC-Eu showed particle size of 71.800 ± 2.144 nm, poly-dispersity index of 0.258 ± 0.003, and zeta potential of − 2.927 ± 0.163 mV. Furthermore, the TEM results of NLC-Eu displayed irregular round to spherical morphology with narrow size distribution and relatively uniformed particles. The drug loading capacity and entrapment efficiency of NLC-Eu were 4.99 and 90.93%, respectively. Furthermore, NLC-Eu exhibited cytotoxic effects on both, human and mice, breast cancer cells with IC50 values of 10.00 ± 4.81 μg/mL and 17.70 ± 0.57 μg/mL, respectively at 72 h. NLC-Eu also induced apoptosis on the MDA MB-231 cells. In the sub-chronic toxicity study, all of the studied mice did not show any signs of toxicity, abnormality or mortality. Besides that, no significant changes were observed in the body weight, internal organ index, hepatic and renal histopathology, serum biochemistry, nitric oxide and malondialdehyde contents.

**Conclusions:**

This study suggests that the well-characterized NLC-Eu offers a safe and promising carrier system which has cytotoxic effect on breast cancer cell lines.

## Background

Naturally derived active compounds and essential oils have been in the spotlight for several years. For instance, zerumbone, curcumin, thymoquinone, citral, thymol, carvacrol, and eucalyptol have been earning the reputation of promising potential therapies for many human ailments especially malignant cancers. Eucalyptol (C_10_H_18_O), or 1,8-cineole, is a natural constituent of a number of aromatic plants and their essential oils such as plants belong to the genera of *Eucalyptus*, *Croton*, *Hyptis*, *Pectis*, *Rosamarinus* and *Salvia* [1], and it is called eucalyptol due to its natural abundance in *Eucalyptus* [2]. Eucalyptol is approved by the Food and Drug Administration (FDA) for human use (cosmetics, aromatherapy and dentistry) and consumption (flavoring agent) [3].

Eucalyptol has the ability to express various biological effects such as antimicrobial [4], anti-hyperglycemic [5], antioxidant [6], analgesic, anti-inflammatory, antiallergic [7], antipyretic [8], anti-tumor [9], gastroprotective [10], and hepatoprotective [11] actions. It was also confirmed that eucalyptol relaxes bronchial and vascular smooth muscle [12], reduced bronchitis, sinusitis, chronic rhinitis, and asthma [13]. Moreover, eucalyptol depresses force development, acts as a calcium channel blocker in reducing contractile activity in rat cardiac muscle [12], as well as increases cerebral blood flow after prolonged inhalation [14]. Eucalyptol also had been reported to have the ability to suppress the production and synthesis of tumor necrosis factor-α (TNF-α), interleukin-1β (IL-1β), leukotriene B4, and thromboxane B2 in inflammatory cells [13]. Previous studies proved that Eucalyptol have the capability to inhibit the growth of breast cancer cells, MCF7, MDA MB-231 and T47D [15, 16]. However, it has low solubility in aqueous solutions which contributes to poor absorption and bioavailability that, subsequently, limits its cytotoxic efficacy.

The application of nanoparticles in medical research has fundamentally revolutionized the specificity of anticancer drugs which are frequently associated with therapeutic side effects. One of the goals for nanoparticles in pharmaceutics is to advance the system for more effective delivery of anti-cancer therapy and diagnosis [17].

Nanostructured lipid carrier (NLC) is a novel nano-carrier platform for the development of effective drug delivery for cancer chemotherapeutics [18]. NLC has been previously reported to improve bioavailability and solubility in most of the hydrophobic cancer drugs, including tamoxifen [19]. NLC is considered as a biodegradable lipid-based nanoparticle composed of solid lipids and liquid lipids in its formulation. In addition, NLC confers controlled drug release properties and increased chemical stability of the incorporated compound because of its highly unordered lipid matrices, where a high stacking of compounds is achieved [20]. Optimally, incorporation of anticancer agents within NLC has been pursued to increase their efficacy while reducing the dose related toxicity [21]. Thus, with this information, the cytotoxicity of the encapsulated eucalyptol was analyzed to study whether the nanoencapsulation exhibit retained or enhanced effects on breast cancer cells in comparison to eucalyptol.

According to previous studies, antitumor study on animal models is important as the effect and delivery of nano samples may not be seen in in vitro study as the improved delivery, mechanism and effect of the nanocarrier of samples is exhibited better in in vivo studies [22–24]. However, prior to studying the effect of NLC-Eu as a potential antitumor agent in tumor-induced animal model, it is required to exhibit good safety profiles [25]. With respect to this, toxicity validation is imperative to measure any possible detrimental reaction on vital organs upon administration of NLC-Eu. After robust confirmation of toxic-free nature of NLC-Eu nano-samples by sub-chronic toxicity study on healthy BALB/c mice is obtained, further in vivo cytotoxicity and biocompatibility analysis with molecular analysis can be carried out in breast cancer-induced BALB/c mice.

In this study, the physicochemical characterization and in vitro cytotoxicity of the nano-encapsulated Eucalyptol (NLC-Eu) through MTT screening towards breast cancer cells, MDA MB-231 and 4 T1, and cell death assessment using dual-staining method were executed. Results of these assessments presented important insight into the potential of the NLC-Eu as an anticancer agent. A systemic toxicity study on NLC-Eu using 28-day oral toxicity following the OECD guidelines was also incorporated as part of its pre-clinical study. The obtained results will assure the suitability and safety of the nano-sample compound and support the potential of NLC-Eu as a safe anti-cancer agent. It would also provide some preliminary data on the safe dosage for NLC-Eu consumption which is an important aspect to be considered for the safe use of NLC-Eu in human.

## Methods

### Materials

The chemicals and reagents used in preparing Nanostructured Lipid Carrier (NLC) in this study are of analytical grade. These included hydrogenated palm oil (HPO) (Sigma Aldrich, USA) that was a gift received from the Institute of Bioscience, Universiti Putra Malaysia (UPM). Eucalyptol 99% (0.921 g/mL at 25 °C) from Sigma-Aldrich (St. Louis, MO, USA), Tween 80 (Merck KGaA, Germany), Lipoid S-100 (Merck Millipore, Germany), olive oil (Basso, Italy), Thimerosal (Sigma, USA) and D-Sorbitol (Sigma, USA). Dulbecco’s Modified Eagle’s Medium (DMEM) and Roswell Park Memorial Institute (RPMI)-1640 Medium were purchased from Sigma (St. Louis, MO, USA), Fetal Bovine Serum (FBS), Tryple™ Express and penicillin-streptomycin from Life Technologies (Carlsbad, CA, USA), while 3-(4,5-dimethylthiazol-2-yl)-2,5-diphenyl tetrazolium bromide (MTT) reagent was purchased from Merck KGaA, Germany.

### Cell culture and cell lines

The cell lines used are human breast cancer cell lines MDA MB-231 (ATCC, HTB-26) and murine breast cancer cell line (4 T1). Both were purchased from the American Type Cell Culture Collection (ATCC, Maryland, USA). MDA MB-231 cells were maintained in Dulbecco’s Modified Eagle Medium (DMEM), while the 4 T1 cells were maintained in a complete growing media of RPMI-1640 (Sigma-Aldrich, USA). Complete growth media were supplemented with 10% fetal bovine serum (FBS) and 1% penicillin-streptomycin (P/S) (Life Technologies, Carlsbad, CA, USA).

### Statistical analysis

All experiments were done in triplicates and the average values were obtained. The statistical analyses were performed using the Statistical Package for the Social Sciences (SPSS) version 22. One-way ANOVA was selected for the experimental analysis with Tukey’s post-hoc test. The significance was set at *p* < 0.05 when comparing to NLC-Blank as a control and NLC-Eu and eucalyptol as treated groups.

### Preparation of nanostructure lipid carrier loaded with eucalyptol

Eucalyptol was loaded into NLC by high-pressure homogenization technique using HPO, Lipoid S-100, olive oil, thimerosal, Tween-80, D-Sorbitol and eucalyptol (99%). The method used to prepare NLC-Eu was similar but slightly modified from previous study [26, 27]. The formulation consisted of 2 phases; lipid phase and aqueous phase. The lipid phase consisting of 4 g of HPO, 1 mL of olive oil, and 1.77 g of Lipoid S-100 were mixed and heated in a beaker at 70 °C. Then, 500 μL of eucalyptol was added to the mixture with constant stirring at 1000 rpm for 5 min. On the other hand, the aqueous phase, which contained 0.005% (w/v) thimerosal, 4.75% (w/v) D-Sorbitol and 2% (w/v) Tween 80, were dissolved and heated at 70 °C. The aqueous phase was then added into the lipid phase under constant stirring for 5 min. The mixture was then further mixed using Ultra-Turrax® (IKA, Staufen, Germany) for 10 min at 13,000 rpm. The emulsion was then pressurized using a high-pressure homogenizer (Avestin, Ottawa, ON, Canada) for 15 cycles at 70 °C. The clear nano-emulsion was then allowed to cool down to room temperature at 25 °C and sealed for 24 h. The NLC-Blank was also formulated using the same method without the addition of eucalyptol during the preparation of lipid phase.

### Characterization of nanostructure lipid carrier loaded eucalyptol

The parameters analyzed for the characterizations of NLC-Eu to determine the quality of the formulation were particle size (PS), polydispersity index (PDI), zeta potential (ZP), transmission electron microscopy (TEM), entrapment efficiency (EE), and drug loading capacity (DLC). Other than that, the stability of NLC-Eu was also assessed over 28 days of duration and lyophilized into solid form.

#### Particle size and polydispersity index

Dynamic light scattering (DLS) method using the Zetasizer Nano ZS (Malvern Instrument, Germany) was used to analyze the average particle size (PS) and polydispersity index (PDI) of NLC-Eu. The formulation was loaded into the folded capillary cuvette and measurement was taken at 25 °C. Three measurements of both were recorded and calculated to obtain the average PS and PDI.

#### Zeta potential

Zetasizer Nano ZS (Malvern Instrument, Germany) was also used to measure the ZP value of the electrophoretic NLC-Eu nano-emulsion. ZP measures the magnitude charges between the particles and is used to predict the long-term stability of the sample as it indirectly measures the diffusion layer of the nanoparticles thickness. NLC-Eu was loaded into the folded capillary cuvette and the readings of ZP were obtained in triplicates.

#### Transmission electron microscopy

It is used to observe the size and shape of the nanoparticles. Briefly, a drop of NLC-Eu was placed on the carbon-coated copper grid. The sample was left to air dry for about 5 min. Negative staining using 2% (w/v) phosphotungstic acid was done on the sample for 1 min and allowed to dry at room temperature. NLC-Eu was then viewed using the transmission electron microscope (Hitachi, Japan) [27, 28].

#### Lyophilization of nanostructured lipid carrier loaded eucalyptol

Lyophilization of NLC-Eu ensures long term storage of the samples as it enhances the chemical and physical stability of the nanoparticles. Lyophilized NLC-Eu permits the incorporation of these nanoparticles into more stable form, such as capsules and tablets. Lyophilized NLC-Eu was prepared using a freeze dryer (Martin Christ GmbH, Osterode am Harz, Germany). Briefly, 5 mL of NLC-Eu nano-emulsion was poured into a plastic petri dish and frozen at − 80 °C (Thermoforma, Marlotta, USA) for a few hours. The plates were then covered using parafilm, which had several holes poked through it, and subsequently, freeze-dried for 24 h at − 55 °C [26].

#### Entrapment efficiency (EE) and drug loading capacity (DLC)

Ultrafiltration method was used to measure the amount of free drug in the sample that enables determination of EE and DLC of the sample. This was performed using Sartorius ultrafiltration centrifugal concentrators, Vivaspin®20 ultrafiltration tubes (Sartorius, Goettigen, Germany) with a molecular weight cut off point of 300 kDa. About 20 mL of NLC-Eu was placed in the upper chamber of the tube and was then centrifuged at 15,000 rpm for 15 min. The principle applied in this method is that the eucalyptol filtered out to the lower chamber is considered free or unbound to the NLC while the eucalyptol remaining in the upper chamber is considered bound to the NLC as NLC-Eu. The free eucalyptol in the lower chamber were then quantified using UV-Vis spectrophotometer (Beckman-Coulter, Fullerton, CA, USA) at 280 nm. Then, the EE and DLC of NLC-Eu were calculated using the equation below [26]:

$$\boldsymbol{EE}\ \left(\%\right)=\frac{\left(\boldsymbol{Total}\ \boldsymbol{amount}\ \boldsymbol{of}\ \boldsymbol{Eucalyptol}\right)-\left(\boldsymbol{Free}\ \boldsymbol{amount}\ \boldsymbol{of}\ \boldsymbol{Eucalyptol}\right)\ }{\left(\boldsymbol{Total}\ \boldsymbol{amount}\ \boldsymbol{of}\ \boldsymbol{Eucalyptol}\right)}$$

$$\boldsymbol{DLC}\ \left(\%\right)=\frac{\left(\boldsymbol{Total}\ \boldsymbol{amount}\ \boldsymbol{of}\ \boldsymbol{Eucalyptol}\ \boldsymbol{encapsulated}\ \boldsymbol{in}\boldsymbol{to}\ \boldsymbol{NLC}\right)\ }{\left(\boldsymbol{Total}\ \boldsymbol{amount}\ \boldsymbol{of}\ \boldsymbol{lipid}\ \boldsymbol{used}\ \boldsymbol{in}\ \boldsymbol{NLC}-\boldsymbol{Eu}\ \boldsymbol{formulation}\right)}$$

#### pH level of nanostructured lipid carrier loaded with eucalyptol

A calibrated S47-K pH meter (Mettler Toledo, Beaumont Leys, UK) was used to determine the pH level of the NLC-Eu at room temperature on the first day of production and also after 21 and 35 days of preparation.

#### Stability assessment of nanostructured lipid carrier loaded with eucalyptol

To monitor the stability of NLC-Eu, sample of the NLC-Eu nano-emulsion was placed in amber tubes directly after preparation and stored at room temperatures. The tubes were then observed for any color, consistency and viscosity changes or any layering appearance by taking pictures at scheduled timepoints. Upon 28 days of monitoring, the changes in appearance of the NLC-Eu sample were recorded.

### In vitro anticancer study

#### MTT assay

MTT assay was conducted to determine the cytotoxicity effects of NLC-Eu, eucalyptol and NLC-Blank treatments on the cells. Briefly, MDA MB-231 and 4 T1 cells were harvested, counted and seeded at 1 × 10^4^ cells per well in the 96-well plate for 24 h. The following day, cells were treated with various concentrations of the sample and incubated for 48 and 72 h at 37 °C with 5% CO_2_. After that, 20 μL of 5 mg/mL MTT (Merck, USA) reagent was added to each well and incubated for 4 h. Next, the solution was removed and 100 μL of dimethyl sulfoxide (DMSO) was added to the wells. Then, the plate was read at 570 nm using the μQuant microtiter plate reader (Bio-Tek Instruments, VT, USA). The results were analyzed as the percentage proliferation of the cells with respect to the concentration of the samples treated.

#### Acridine orange and propidium iodide (AO/PI) double staining

Acridine orange and propidium iodide (AO/PI) double staining is used to determine the morphological changes of MDA-MB-231 cells upon treatment with NLC-Eu. Cells were seeded in 6-well plates and were incubated overnight. The next day, the cells were treated with 2 inhibitory concentrations (IC) of NLC-Eu; IC25 and IC50 for 48 h. Afterwards, the cells were trypsinized and washed with PBS twice. The harvested cells were resuspended in 100 μL PBS and stained with 10 μg/mL AO and PI dyes. AO is permeable and stains DNA within viable cells directly while emitting green fluorescence once it is excited [29]. PI, on the other hand, is impermeable to the cell membrane of viable cells and can only bind to DNA when the membrane is compromised while emitting a red to orange fluorescence. It was then viewed under a fluorescence microscope (Nikon, Japan).

### In vivo sub-chronic toxicity study

#### Animals

A total of 24 male adult BALB/c mice aged 6 to 8 weeks weighted 20-25 g were purchased from the Animal House of Faculty of Veterinary, Universiti Putra Malaysia, Serdang, Selangor, Malaysia. The mice were acclimatized for 1 week and monitored under 12-h dark and light cycle under the standard condition at 2 ± 1 °C. The mice were provided water and pellet ad libitum during the whole period of study. This study was approved by the Institutional Animal Care and Use Committee (IACUC), Faculty of Veterinary Medicine, Universiti Putra Malaysia (UPM/IACUC/AUP-R024/2018).

#### Animals grouping and treatments

The mice were randomly grouped into 4 groups (*n* = 6): (1) NLC-Blank treated group, (2) NLC-Eu treated group, and (3) Eucalyptol treated group (4) Untreated group. Mice in all four groups were treated orally with the treatments of 50 mg/kg of NLC-Eu, 50 mg/kg of NLC-Blank, 50 mg/kg of eucalyptol and distilled water daily using oral gavage for 28 days [30].

#### Clinical observations

During the oral administration of the NLC-Eu nano formulation, the animals were observed for clinical abnormalities twice a day (morning and afternoon) for the onset of clinical or toxicological symptoms. Mortality (if any), signs of toxicity, body weight, food intake or consumption and gross findings were observed and noted over the period of 28 days of treatment.

#### Body weights changes

Individual body weights (BW) the test animals were obtained prior to dosing on days 0, 7, 14, 21, and 28 using a sensitive balance ER120-A (A&D Company, Limited, Japan).

#### Organ-to-body-weight index

On the 28th day, the mice were sacrificed by terminal exsanguination under anesthesia (100 mg/kg Ketamine – 10 mg/kg xylazine per kg body weight). Vital organs such as heart, kidneys, liver, lung and spleen were autopsied and examined macroscopically for any lesions or abnormalities. Body weight and weight of the organs from the untreated control, NLC-Eu, NLC-Blank and eucalyptol treatment groups were measured and recorded. The relative organ weight of each animal was then calculated as follows [31]:

$$\mathbf{Relative}\ \mathbf{organ}\ \mathbf{weight}=\frac{\mathbf{Absolute}\ \mathbf{organ}\ \mathbf{weight}\ }{\mathbf{Body}\ \mathbf{weight}\ \mathbf{of}\ \mathbf{mice}\ \mathbf{on}\ \mathbf{Day}\ \mathbf{28}}\times \mathbf{100}\%$$

All the individual organs were macroscopically observed and the appearances were compared between treated and control groups. Statistical analysis to assess the significant difference between both groups was conducted by running independent sample t-test using SPSS (version 22.0) spreadsheet application. The level of significance used in this analysis was 5%.

#### Histopathology of kidney and liver

Liver and kidney samples were cut into sections of about 0.5 cm^2^ and preserved in 10% formalin for 48 h. The preserved samples were placed in plastic cassettes and dehydrated using an automated tissue processor (Leica ASP300, Germany). The processed tissues were then embedded in paraffin wax (Leica EG1160, Germany). The blocks were trimmed and sectioned to about 5 × 5 × 4 μm size using a microtome (Leica, RM2155). The tissue sections were mounted on glass slides using a hot plate (Leica HI1220, Germany) and then treated in order with 100, 90 and 70% ethanol for 2 min each. Finally, the tissue sections were rinsed with tap water and stained with the hematoxylin and eosin (H&E) and examined under a light microscope (Nikon, Japan).

#### Serum biochemistry

Blood samples were collected in a plain tube from the mice by cardiac puncture. The serum was obtained by centrifugation at 3000 rpm for 15 min. The concentration of aspartate aminotransferase (AST), alanine aminotransferase (ALT), alkaline phosphatase (ALP), total bilirubin, creatinine, urea and triglyceride level in mice serum were analyzed accordingly using Hitachi automatic analyzer (Hitachi-902, LTD, JAPAN).

#### In vivo nitric oxide detection

Nitric Oxide (NO) detection from the liver and kidneys was established with the Griess Reagent Kit (Sigma, USA). The experiment was conducted according to the protocol provided with the kit. The supernatant from the splenocytes was mixed with Griess Reagent and incubated for 30 min before being measured at 548 nm using microplate reader (Beckman Coulter, USA).

#### Malondialdehyde detection

The malondialdehyde (MDA) content is an indicator of lipid peroxidation in the vital organs of animal samples. The level of lipid peroxidation from untreated and treated groups was assayed by thiobarbituric acid (TBA) reactive substance by the method of Garcia, Rodriguez-Malaver & Penaloza [32]. Briefly, the liver and kidney were harvested, meshed and filtered with the 70 μm cell strainers in PBS before centrifuged at 2000 rpm for 5 min. Then, 200 μL of supernatant was mixed with 800 μL of PBS, 25 μL of the 8.8% of butylated hydroxytoluene (BHT) and 500 μL of 30% of trichloroacetic acid (TCA), followed by incubation on ice for 2 h. After that, the mixtures were centrifuged at 2000 rpm and mixed with 250 μl of 1% thiobarbituric acid (TCA) and 75 μL of EDTA before they were heated to 95 °C for 15 min. Next, the mixture was chilled to room temperature and the absorbance was read at 532 nm using a spectrophotometer (Beckman Coulter, USA). The standard was prepared using 1,1,3,3-tetramethoxypropane (Sigma Aldrich, USA). The MDA concentration was calculated based on the standard curve of 1,1,3,3-tetramethoxypropane.

## Results

### Preparation of nanostructured lipid carrier loaded with eucalyptol

**Fig. 1:**
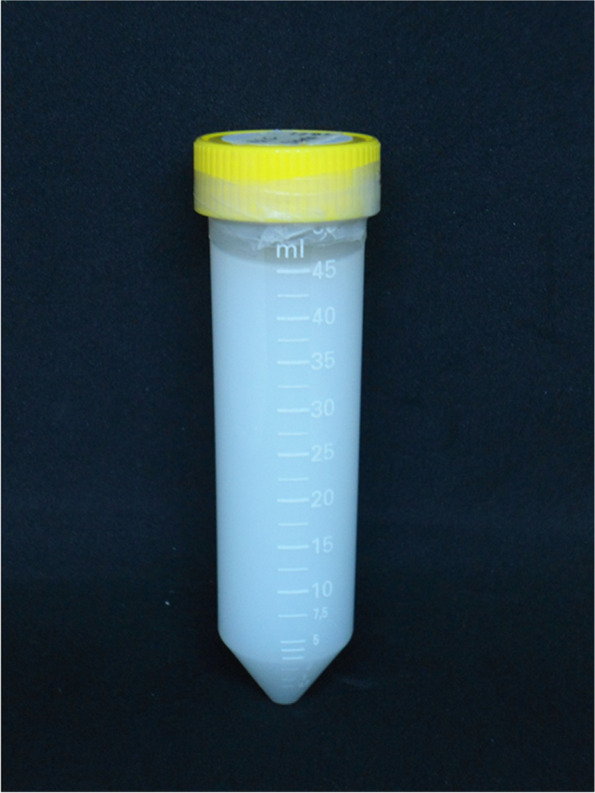
Eucalyptol-loaded Nanostructured Lipid Carrier (NLC-Eu)

Translucent, light milky colored active compound-loaded NLC-Eu was prepared using high pressure homogenization technique (Fig. 1).

### Physicochemical characterization of nanostructure lipid carrier loaded eucalyptol

Characterizations of NLC-Eu were done by determining the PS, PDI, ZP, morphology, EE, DLC, pH, and stability under storage condition of room temperature. Microscopical images revealed that NLC-Eu possessed nano-sized particles, which correlates with the data obtained using dynamic light scattering method.

#### Particle size and polydispersity index

**Fig. 2:**
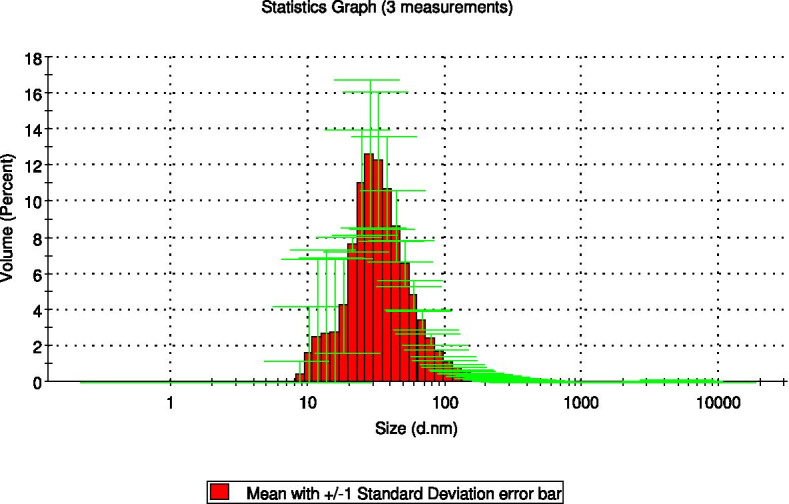
The particle size distribution of Nanostructured Lipid Carrier loaded with Eucalyptol (NLC-Eu) obtained from the Malvern Instrument Report

The average PS and PDI are especially crucial characteristics for nanoparticle samples as they determine the feature and stability of the NLC when the active compound is loaded into these nanocarrier sample. The average PS and PDI of NLC-Eu were 71.800 ± 2.144 nm (Fig. 2) and 0.258 ± 0.003, respectively.

#### Zeta potential

In order to determine the overall stability of the NLC-Eu nano-formulation, obtaining the ZP is necessary. The surface charge analysis or average ZP of NLC-Eu is − 2.927 ± 0.163 mV. The summary of physicochemical characteristics of NLC-Eu formulation is shown in Table 1.

**Table 1:** The characterization of NLC- Eucalyptol and NLC-Blank

**Characterization**	**NLC-Eucalyptol**	**NLC-Blank**
Particle size	71.800 ± 2.144 nm	108.400 ± 1.217 nm
Zeta potential	−2.927 ± 0.163 mV	- 2.813 ± 0.085 mV
Polydispersity index	0.258 ± 0.003	0.598 ± 0.006

All data are expressed as mean ± SD

#### Transmission electron microscopy

**Fig. 3:**
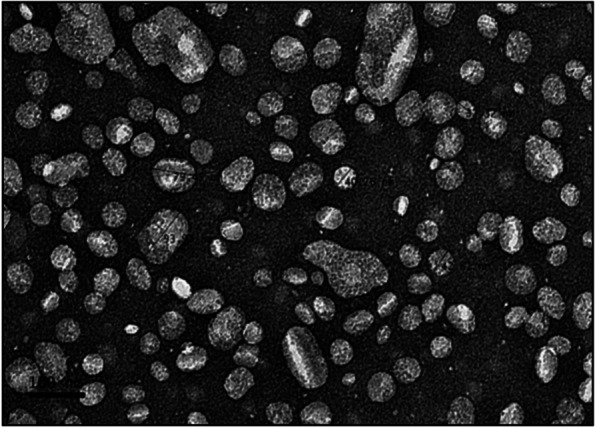
Transmission electron microscopy (TEM) image of Nanostructured Lipid Carrier loaded with Eucalyptol (NLC-Eu) at 800,000× magnification prepared by negative staining

Figure 3 shows the TEM image of NLC-Eu that appeared as irregular round to spherical-shaped, narrow size distribution and relatively uniformed particles. The average particle size distribution of NLC-Eu from TEM image was 74.80 ± 2.31 nm and considered within the same range as reported from the Zetasizer Nano ZS reading.

#### Lyophilization of nanostructured lipid carrier loaded with eucalyptol

**Fig. 4:**
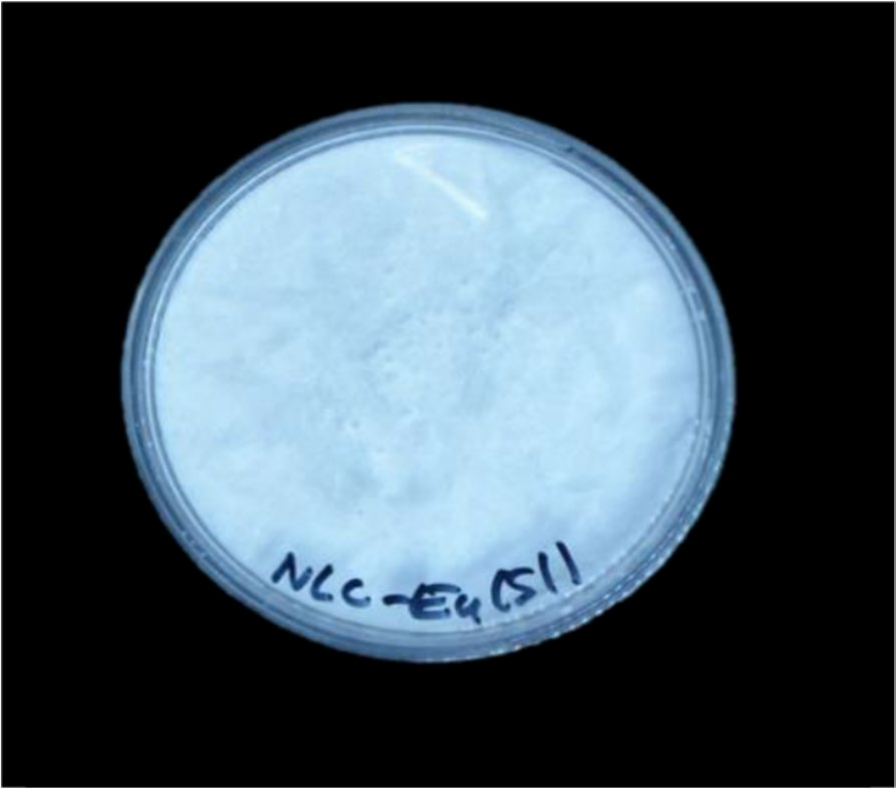
Lyophilized nanostructured lipid carrier loaded with eucalyptol (NLC-Eu)

Lyophilized NLC-Eu was freeze dried for 48 h and the final product appeared as a white powder with fibrous texture. Figure 4 shows the end product of lyophilized NLC-Eu.

#### Drug loading capacity and entrapment efficiency

This study showed that of the 500 mg of eucalyptol that was used to load into the NLC, approximately 4.35 mg free eucalyptol was detected in aqueous NLC-Eu dispersion. This suggests that 90.93% w/w (495.65 mg) of eucalyptol were successfully encapsulated into the NLC. Thus, EE and DLC of NLC-Eu were 90.93 and 4.99%, respectively (Table 2).

**Table 2:** The drug loading capacity and entrapment efficiency of Nanostructured Lipid Carrier loaded with Eucalyptol (NLC-Eu)

**Characterization**	**NLC-Eucalyptol**
Drug loading capacity	4.99 ± 0.14%
Entrapment efficiency	90.93 ± 0.39%

All data are expressed as mean ± SD

#### pH level of nanostructured lipid carrier loaded with eucalyptol

The pH of NLC-Eu formulations upon storage at room temperature was observed to have increased from 2.90 to 3.04 and 3.12 at week 1, 3 and 5 post-preparations of NLC-Eu, respectively (Table 3).

**Table 3:** pH level of Nanostructured Lipid Carrier loaded with Eucalyptol (NLC-Eu)

**Time**	**pH level**
Day 01 (week 1)	2.90
Day 21 (week 3)	3.04
Day 35 (week 5)	3.12

#### Stability assessment of nanostructured lipid carrier loaded with eucalyptol

**Fig. 5:**
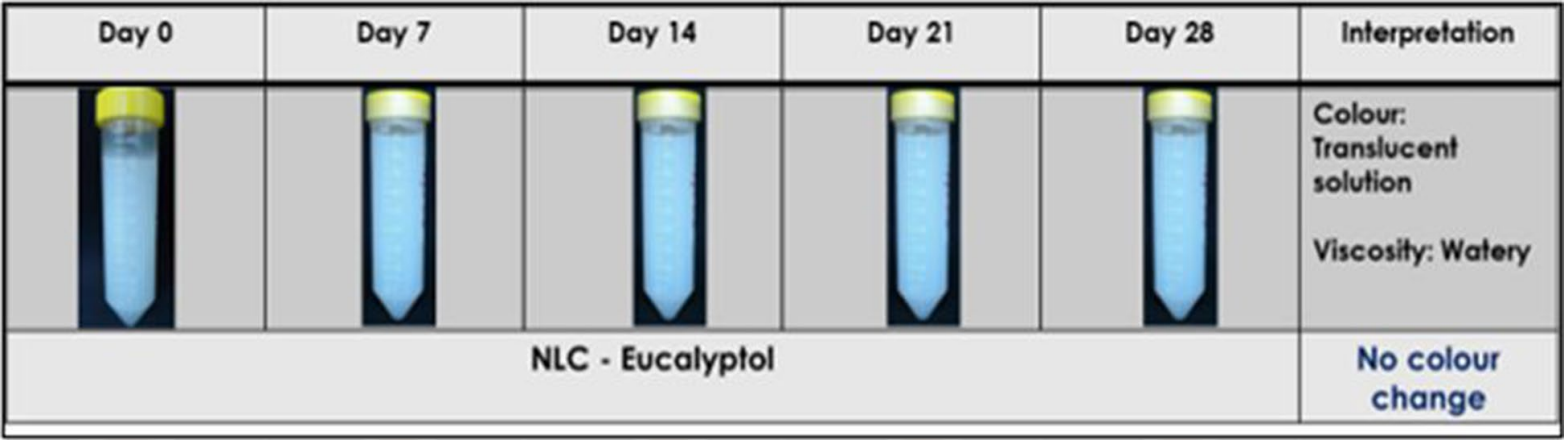
Stability assessment and image of nanostructured lipid carrier loaded Eucalyptol (NLC-Eu) over 28 days observation duration

The stability assessment in terms of optical observation in presence of sedimentation or appearance of oil layer at the top of the formulation was done within 28 days upon preparation of the formulation (Fig. 5). Besides that, the stability assessment in terms of PS, PDI, and ZP of NLC-Eu were analyzed at 6-month post-formulation to determine whether the nanoparticles remained the same or agglomerate over time. Table 4 depicts the changes to the physicochemical properties of NLC-Eu after 6 months. Generally, there was an increase in PS of NLC-Eu from 71.800 ± 2.144 nm to 87.250 ± 0.102 nm, a slight increase in PDI from 0.258 ± 0.003 to 0.380 ± 0.001, as well as ZP from − 2.927 ± 0.163 mV to − 4.271 ± 0.123 mV 6 months post-preparation. These small changes indicated that the nanoparticles may become bigger and slightly agglomerate over time.

**Table 4:** The stability assessments of nanostructured lipid carrier loaded Eucalyptol (NLC-Eu)

**Characterization**	**Day 0**	**6 Months Post-preparation**
Particle size	71.800 ± 2.144 nm	87.250 ± 0.102 nm
Zeta potential	−2.927 ± 0.163 mV	−4.271 ± 0.123 mV
Polydispersity index	0.258 ± 0.003	0.380 ± 0.001

All data are expressed as mean ± SD

### In vitro anticancer study

#### NLC-Eucalyptol reduces viability of human breast cancer, MDA MB-231 and mouse breast cancer, 4 T1 cells

The MTT assay was used to evaluate the anti-proliferative effects of NLC-Eu and eucalyptol on human breast cancer cell line, MDA-MB-231, and mouse breast cancer cell line, 4 T1. The cells were treated with two-fold serial dilutions of NLC-Eu and eucalyptol for up to 72 h. The half-maximal inhibitory concentration (IC50) of NLC-Eu on MDA-MB-231 and 4 T1 cells was significantly lower than that of eucalyptol. For both time points, the NLC-Eu treatment proved to be more than twice as effective to inhibit MDA-MB-231 cell proliferation as compared to eucalyptol (Table 5). In the mouse breast cancer cells, IC50 of eucalyptol at 48 h and 72 h time points were higher at almost four times the IC50 of NLC-Eu, signifying its enhanced effectiveness when encapsulated (Table 6).

**Table 5:** The IC50 value of NLC-Eu and Eucalyptol on human breast cancer cell line, MDA MB-231

**Time point**	**Half-maximal inhibitory concentrations (μg/mL)**		
	**NLC-Eucalyptol**	**Eucalyptol**	**NLC-Blank**
48H	11.50 ± 3.68*	25.00 ± 5.45	19.80 ± 5.52
72H	10.00 ± 4.81*	22.00 ± 9.12	19.00 ± 13.36

All data are expressed as mean ± SD

* indicate *p* < 0.05 compared to eucalyptol

**Table 6:** The IC50 value of NLC-Eu and Eucalyptol on mouse breast cancer cell line, 4 T1

**Time point**	**Half-maximal inhibitory concentrations (μg/mL)**		
	**NLC-Eucalyptol**	**Eucalyptol**	**NLC-Blank**
48H	17.80 ± 0.29*	68.50 ± 1.63	35.40 ± 0.28
72H	17.70 ± 0.57*	65.70 ± 5.63	24.90 ± 8.56

All data are expressed as mean ± SD

* indicate *p* < 0.05 compared to eucalyptol

#### NLC-Eu treatment induced apoptosis in MDA-MB-231 cells

**Fig. 6:**
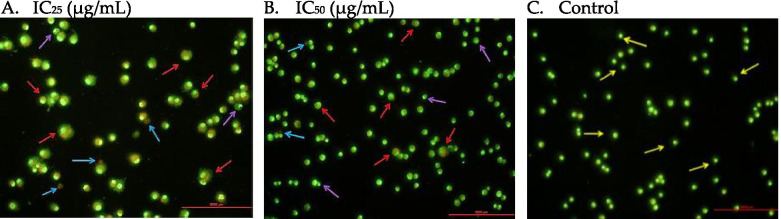
Micrograph of MDA-MB-231 cells treated with **A** IC25 concentration of NLC-Eu at 48 h; early apoptosis: 66.90%, late apoptosis: 30.28% and necrosis: 2.82%, **B** IC50 concentration of NLC-Eu at 48 h timepoint; early apoptosis: 52.38%, late apoptosis: 42.86% and necrosis: 4.76%, and **C** Control. Arrows indicate cells at different stages namely viable (yellow), early apoptosis (purple) and late apoptosis (red). Blue arrows indicate necrotic cells

NLC-Eu proclaimed better cytotoxicity effect on human breast cancer cell line MDA MB-231 as compared to mouse breast cancer cell line, 4 T1. To examine the cell death pathway on the human breast cancer cells, AO/PI dual-staining assay was incorporated into this study. The assay enables morphology and cellular profiles for viable, apoptotic and necrotic cells to be distinguished. As the viable and healthy cells have regular size and intact cell membrane, these cells appear bright green in the micrograph. Early apoptotic cells also fluoresce green but can be distinguished with the appearance of membrane disruption, also known as membrane blebbing, and nuclear condensation within the individual cells. On the other hand, late apoptotic cells showed distinct occurrences of nuclear distortion, apoptotic bodies, appear enlarged and are red stained within the cells due to the heavily compromised cell membrane while necrotic cells fluoresce red. Based on Fig. 6A and B, the IC25 (7.8 ± 1.28 μg/mL) and IC50 (11.50 ± 3.68 μg/mL) treatments of NLC-Eu induced apoptosis as indicated by the purple and red arrows where increased amount of cells undergoing late apoptosis and early apoptosis were observed in the cell population. Figure 6C depicts the viable and healthy MDA-MB-231 cells which were cultured without any treatment using complete growth medium for 48 h.

### In vivo sub-chronic toxicity study

#### Animals and sub-chronic toxicity study

Sub-chronic toxicity study using animal models is essential to examine the in vivo toxicity of a formulation. This was performed to evaluate the adverse effect of NLC-Eu formulations when administered at a dosage of 50 mg/kg BW to BALB/c mice via oral consumption for 28 days [30]. The dosage was chosen based on a previous study by Nordin et al. [30].

#### Clinical observations

Changes and physical abnormalities were observed in the mice upon treatment with NLC-Eu, Eucalyptol and NLC-Blank. The clinical observation and abnormalities attributes which were being observed include the posture and movement of the BALB/c mice, the coat condition; well groomed, and also their eating, drinking and breathing behaviors. There were no signs of toxicity or abnormality in any mice.

#### Mortality and body weights changes

**Fig. 7:**
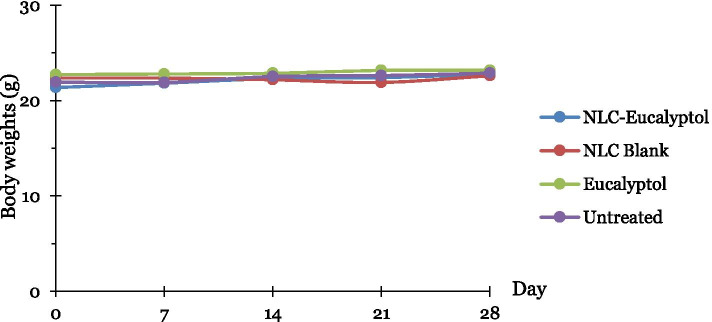
Body weight of mice from each group, NLC-Eucalyptol, NLC-Blank, Eucalyptol and Untreated group at during 28 days of sub-chronic toxicity study treatment at day 0, day 7, day 14, day 21 and day 28

All 24 BALB/c mice treated with NLC-Eu, eucalyptol, NLC-Blank and the untreated mice survived till 28 days of treatment. According to Table 7, there was no mortality, body weight changes and no conspicuous signs of toxicity rises throughout the sub-chronic toxicity study (Fig. 7).

**Table 7:** The observation of mortality, body weight changes and toxicity signs of NLC-Eucalyptol, NLC-Blank, Eucalyptol and Untreated groups

**Treatment groups**	**Mortality at 28th days**	**Body weight changes**	**Toxic signs**
**NLC-Eucalyptol**	None	None	None
**NLC-Blank (vehicle)**	None	None	None
**Eucalyptol**	None	None	None
**Untreated (control)**	None	None	None

#### Organ index

In agreement with the absence of clinical abnormalities in all mice, the organ index of liver, spleen, lungs, heart and kidney of the mice treated with 50 mg/kg NLC-Eu group and 50 mg/kg eucalyptol did not statistically differ when compared to the control mice group (untreated) (Table 8).

**Table 8:** The organ index (absolute organ weight divided by body weight of mice on day 28) of liver, spleen, lungs, heart and kidney of BALB/c mice treatment groups

**Treatment groups**	**Liver**	**Spleen**	**Lungs**	**Heart**	**Kidneys**
NLC-Eu	4.30 ± 0.41	0.26 ± 0.04	0.54 ± 0.03	0.50 ± 0.04	1.70 ± 0.05
NLC-Blank	4.00 ± 0.33	0.29 ± 0.04	0.62 ± 0.01	0.52 ± 0.01	1.50 ± 0.06
Eucalyptol	4.52 ± 0.07	0.25 ± 0.02	0.61 ± 0.19	0.50 ± 0.10	1.55 ± 0.20
Untreated	4.33 ± 0.09	0.31 ± 0.07	0.59 ± 0.05	0.57 ± 0.03	1.64 ± 0.06

All data are expressed as mean ± SD

#### Histopathology of kidney and liver

**Fig. 8:**
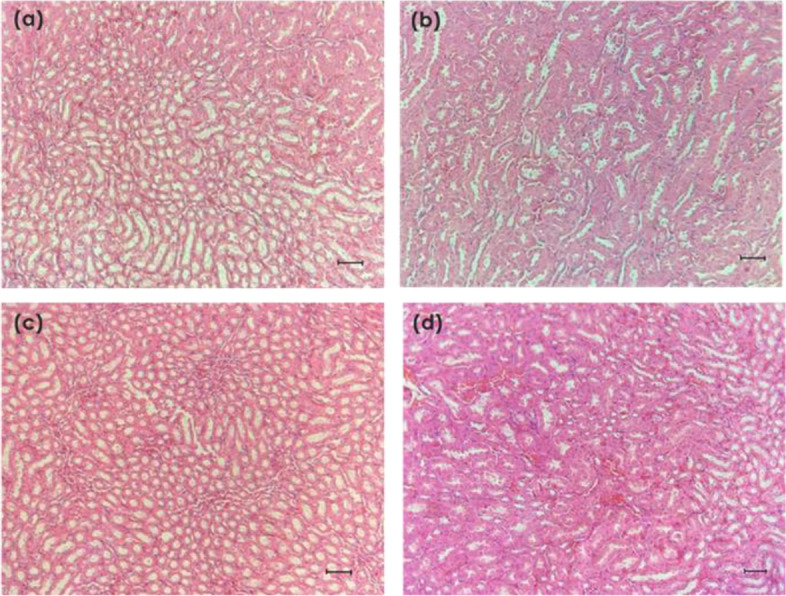
Histology images of BALB/c mice kidneys in groups: **a** NLC-Eucalyptol treated group, **b** NLC-Blank treated group, **c** Eucalyptol treated group and **d** Untreated group, H&E, formalin-fixed tissue section, × 200. Bar = 2000 μm

Based on the histopathology images obtained, the kidneys of the mice from all treatment groups appeared to be normal. The epithelial lining of the tubules and glomerular architecture in all treatment groups of NLC-Eu, NLC-Blank and eucalyptol appeared normal when compared to the control group (Fig. 8).

**Fig. 9:**
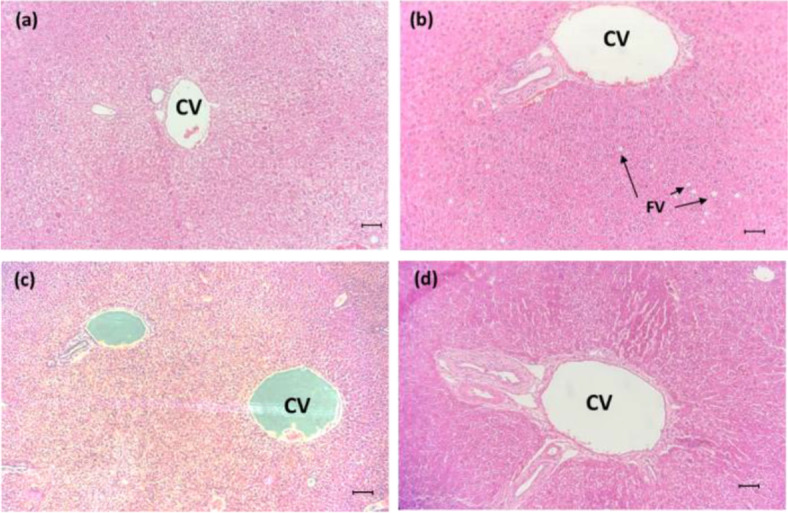
Liver histology images of BALB/c mice groups: **a** NLC-Eucalyptol treated group, **b** NLC-Blank treated group, **c** Eucalyptol treated group and **d** Untreated group, H&E, formalin-fixed tissue section, × 200. Bar = 2000 μm. (CV: Central vein, FV: Fat vacuoles)

Under microscopic examination, the liver in majority of test animals showed normal cellular architecture with most of the central veins (CV) and hepatocytes are still arranged in cords with presence of binucleated formation. Other than that, examinations of liver sections obtained from the groups treated with NLC-Eu and Eucalyptol for 28 days were similar to the untreated group and showed normal histological structure of the liver with polygonal shaped hepatocytes containing rounded nuclei. In hepatocytes of the NLC-Blank treatment group, occurrence of mild hepatic lipidosis or microvesicular hepatic steatosis, in the form of small fat vacuoles (FV), was observed in the liver cross-section. There were no signs of injury or hemorrhage to the central veins in all treatment groups (Fig. 9).

#### Serum biochemistry

Collected blood was used for the estimation of serum biochemical parameters. After 28 days of treatment, no significant (*P* > 0.05) alterations were observed in all hepatic serum liver function biochemical parameters in animals of NLC-Eu treated groups (50 mg/kg) compared to the control group (Table 9). There were non-significant elevations of AST and ALT levels observed in the serum profile of NLC-Blank treatment group compared to the untreated group. This may have resulted from the disposition of fats as seen in the histology image (Fig. 9b).

**Table 9:** The serum biochemical analysis results of NLC-Eu, NLC-Blank, Eucalyptol and untreated group

**Treatment groups**	**ALP (U/L)**	**AST (U/L)**	**ALT (U/L)**	**Total Bilirubin (umol/L)**	**Creatinine (umol/L)**	**Triglyceride (mmol/L)**
NLC-Eu	63.50 ± 11.12	679.5 ± 183.18	64.50 ± 12.79	2.25 ± 0.34	18.50 ± 5.97	1.38 ± 0.66
NLC-Blank	70.00 ± 16.41	1202.50 ± 159.37	144.00 ± 68.37	2.10 ± 1.31	21.50 ± 1.00	1.45 ± 0.21
Eucalyptol	67.50 ± 10.30	679.50 ± 135.04	71.50 ± 12.15	2.30 ± 0.38	19.50 ± 4.43	1.52 ± 0.56
Untreated	67.33 ± 2.31	791.33 ± 179.08	80.67 ± 4.16	2.07 ± 0.42	20.00 ± 4.00	1.28 ± 0.12

Values represent means and standard deviation

#### In vivo nitric oxide detection

**Fig. 10:**
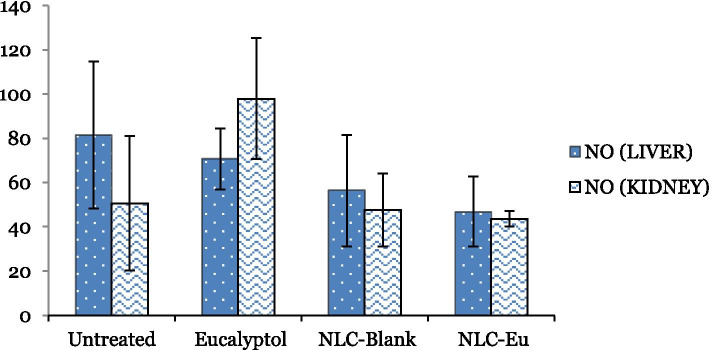
Bar chart analysis of the level of nitric oxide (NO) detected in BALB/c mice’s liver and kidney harvested from the NLC-Eu, NLC-Blank, Eucalyptol treated groups and untreated group after 28 days of treatment (50 mg/kg of body weight). Each value represents the means ± standard deviation. Significance was set at *P* < 0.05 comparing between groups

The nitric oxide levels of NLC-Eu, NLC-Blank, Eucalyptol and the untreated treatment groups were evaluated to study the inflammatory effects of each formulation. As illustrated in Fig. 10, all the treatment groups, NLC-Eu, NLC-Blank and eucalyptol, reduced the level of NO when compared to the untreated BALB/c mice treatment group. Compared to the untreated group (81.74 ± 33.25 μM/mg), there were slight reductions of NO levels detected in the liver of the NLC-Eu (46.94 ± 15.88 μM/mg) treated group, eucalyptol (70.66 ± 13.71 μM/mg) treated and NLC-Blank (56.44 ± 25.16 μM/mg) treated mice group, however, these results were not significant. Similarly, there was also no significant reduction of NO level in the NLC-Eu treatment group when compared to eucalyptol treatment group.

#### Malondialdehyde detection

**Fig. 11:**
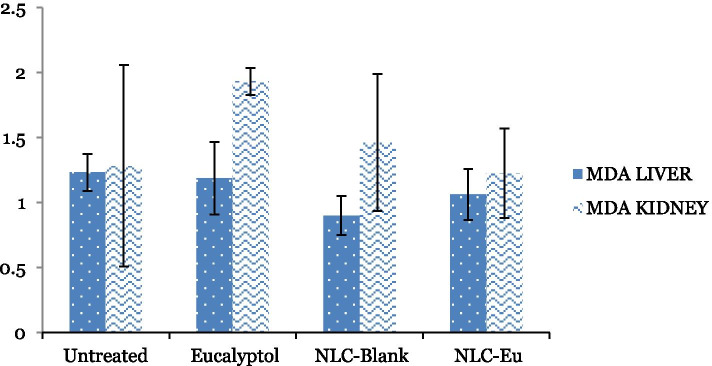
Malondialdehyde levels in BALB/c mice liver and kidneys in NLC-Eu, NLC-Blank, Eucalyptol treated groups and the untreated groups. Each value is represented as mean ± SD, the experiment was done in triplicate. Significance was set at *P* < 0.05 comparing between groups

In the in vivo analysis, the level of lipid peroxidation of MDA in the BALB/c mice treated with NLC-Eu, NLC-Blank, Eucalyptol and the untreated group was measured from the supernatant of liver and kidney. The level of MDA in the liver was slightly reduced to 1.06 ± 0.20 nmol/mg in NLC-Eu treated group from 1.23 ± 0.14 nmol/mg as in the untreated group. A similar pattern was seen in the MDA level in kidney where NLC-Eu treatment reduced the MDA level slightly to 1.23 ± 0.35 nmol/mg as compared to untreated group (1.28 ± 0.77 nmol/mg) (Fig. 11). However, the reduction of MDA levels of NLC-Eu and NLC-Blank were not significant when compared to the untreated and eucalyptol treatment groups.

## Discussion

### Preparation of nanostructured lipid carrier loaded with eucalyptol (NLC-Eu)

NLC carriers offers better drug delivery due to the advantages they possess such as the ability to be loaded with hydrophobic drugs, possess enhanced delivery of lipophilic compounds or drugs and increased drug tolerability upon consumption. These attributes enable these carriers as an efficient solution to solubility issues while serving as a vehicle with enhanced drug loading capacity [33–35]. Colloidal drug carrier systems had received great attention as a potential drug delivery system of insoluble drugs because they have many advantages as drug delivery vehicles by increasing the bioavailability and solubility saturation of the drugs, and also their dissolution rate. In this study, a colorless 99% purity eucalyptol was used to encapsulate in the nanocarrier system, NLC. The NLC-Eu formulation was prepared using high pressure homogenizer which allows for much smaller particle sizes with the aim to increase the surface area and bioavailability of the nanoparticles.

### Physicochemical characterization of nanostructure lipid carrier loaded with eucalyptol

A good nanoparticle formulation has droplet size less than 200 nm with small size distribution between nanoparticles with PDI less than 0.4 [26, 28]. Estimations of PS and PDI of nanoparticles were done as these parameters are important features in predicting the stability of drug loaded NLC [19]. Zeta potential (ZP), or surface charge analysis, is the determination of electrokinetic potential of a colloidal system that indicates the electrostatic strength of repulsion between the particles of NLC-Eu. The significance of ZP value is to access the stability of the colloidal dispersion between the nanoparticles. Generally, a good nano-emulsion ZP value lies greater than + 30 mV or less -30 mV as the particles formulated would have adequate repulsive force, less aggregation due to electrostatic repulsion and thus leading to having a better stability [26–28]. However, several experiments have proven that the stability of nano particle are not fully dependent on the electrostatic repulsion but also via the usage of steric stabilizers during preparation [26]. Surfactants that were used as steric stabilizers during nano formulation enable the formulation to have a better stability of dispersion and could control the particle size of samples [27]. For NLC-Eu preparation, Tween 80 was chosen and used to provide a good steric stabilization. Studies have deduced that nano formulations with low zeta potential are stable enough with the supplementary use of higher molecular weight of surfactant which provides steric stabilization in the nanoparticles [36, 37]. Similarly, previous studies done on Domeperidone loaded with NLC and SLN also used the Tween 80 in the formulation was able to provide a good steric stabilization [38]. The average particle size and polydispersity index of the formulated NLC-Eu are relatively small with little variability in size distribution. While the zeta potential of NLC-Eu obtained is relatively low (− 2.927 ± 0.163 mV), further stability assessment was done to study its constancy within 6 months of formulation.

The study of the morphology of NLC-Eu particles was done within 7 days of preparation. The image obtained confirmed that NLC-Eu was nanometer in size, round to spherical and relatively uniformed in shape with narrow size distribution. The size of nanoparticles observed from micrograph (Fig. 3) was slightly bigger compared to the size of particles from the Zetasizer analysis. This may be due to the differences in the principles that were used in both methods, namely, difference in measuring conditions and the method used. The TEM method of measurement was done by the exposure of high vacuum electron beam column on a static sample or tissue, while the Zetasizer analysis utilizes photon correlation spectroscopy, also known as dynamic light scattering (DLS), and the measure of particle size using the Brownian displacement equation [39].

Lyophilization is the best way to increase physical and chemical stability of the formulated NLC-Eu sample for long term storage by improving the incorporation of the samples, commonly into powder form, or in the form of capsules, pellets and tablets [40]. The stable formulation of NLC-Eu enables longer storage of the prepared sample, thus, can be used for characterization and further assays for this study.

When studying the encapsulation efficiency (EE) and drug loading capacity (DLC) of NLC-Eu, it was seen that when 500 mg of eucalyptol was used to be loaded into the NLC, approximately 4.35 mg of free eucalyptol was detected in the aqueous NLC-Eu dispersion. This suggests that the remaining amount of 495.65 mg of eucalyptol was successfully encapsulated in the NLC system. The drug made must be highly soluble in lipid at melting point to ensure adequate DLC, while to achieve high entrapment efficiency, it is necessary to decrease the lipid concentration. The high EE of NLC-Eu, 90.93% is due to the low water solubility and high lipophilicity of eucalyptol. Better flexibility in the modulation of EE and DLC was usually shown in lipophilic components of liquid lipids rather than solid lipids [41–43]. Thus, the DLC of NLC-Eu of 4.99% observed in this study was due to the high solubility of eucalyptol in olive oil and HPO.

The results for pH value observation of the NLC-Eu formulation indicated that the pH values of 2.90, 3.04 and 3.12 obtained on day 1, week 3 and week 5, respectively, increased when stored at room temperature. The chemical and physical stabilities of the nanosuspension are dependent on the pH value [26]. The pH change observed may be due to the lipid degradation, which would eventually lead to formation of free fatty acid (FFA) and would gradually decrease the pH value [44].

The stability assessment of NLC-Eu showed that the average PS of NLC-Eu increased from 71.800 ± 2.144 nm to 87.250 ± 0.102 nm, the PDI also experience a slight increase from 0.258 ± 0.003 to 0.380 ± 0.001, and the ZP from − 2.927 ± 0.163 mV to − 4.271 ± 0.123 mV, respectively. Usually, a long-term stability of nano-emulsion is evaluated and verified by stability studies conducted over a period of time, for example within the course of 1 to 3 months or up to 6 months. Consequently, the NLC-Eu prepared in this study presents a good short-term stability against agglomeration. This is because after a 6-month storage period, there was only a slight increase detected in the PS, PDI and ZP between Day 0 and 6-month post-preparation analyses. A small difference in the PDI exhibits that the formulation sustained its uniformity and homogeneity between the particles, indicating slight to no agglomeration occurrence, thus, representing a stable formulation over the 6-month duration [28]. There was also a slight change in ZP of NLC-Eu that was stored at room temperature for 6 months which suggests that the NLC-Eu formulation stored at room temperature is physically stable as it did not flocculate or agglomerate [26].

### In vitro anticancer study

Commercialized drugs such as tamoxifen and cisplatin are well-known chemotherapy drugs used to treat breast cancer and reduce the risk of cancer recurrence for breast cancer patients. A previous study reported the IC50 value of tamoxifen and cisplatin towards MDA MB-231 cells are 14.56 μg/mL and 6.93 μg/mL, respectively [45, 46]. Based on the preliminary assessment by MTT assay, the NLC-Eu (11.50 ± 3.68 μg/mL) has showed two-fold enhanced cytotoxicity towards human breast cancer cell, MDA MB-231 when compared to eucalyptol (25.00 ± 5.45 μg/mL). While in the mice breast cancer cells, 4 T1, the treatment of NLC-Eu (17.80 ± 0.29 μg/mL) was four times more potent compared to eucalyptol (68.50 ± 1.63 μg/mL). It was observed that NLC-Eu displayed better cytotoxicity effect on MDA MB-231 cells compared to 4 T1 cells. In comparison, the cytotoxicity of the formulated NLC-Eu towards MDA MB-231 cells is greater than that of tamoxifen, but less potent than cisplatin. However, it is noteworthy that the current chemotherapy using these two drugs alone or in combination has been reported to cause adverse side effects [47]. Further analyses of the cell death mechanism of NLC-Eu towards the human breast cancer cell line was assessed by an apoptosis analysis using AO/PI dual staining on MDA MB-231 cells. The results revealed that the treatment on NLC-Eu induced cell death via apoptosis, instead of necrosis, as shown in Fig. 10, where distinguishable features of apoptosis such as membrane blebbing, nuclear condensation, cell shrinkage and apoptotic bodies can be seen. Necrotic cells would be observable by cells that are stained by the propidium iodide and appear red [48]. Previous reports by Taha et al. and Kahkeshani et al. conceded that essential oil rich in eucalyptol from *Cinnamomum glanduliferum* and *Nepeta menthoides* has significant cytotoxicity against MCF7 and MDA MB-231 human breast cancer cell lines [15, 16]. In agreement, our study further confirmed the potency of eucalyptol as indicated by the better cytotoxicity effect of NLC-Eu nano-formulation on the breast cancer cell lines, indicating its potential as an anti-breast cancer agent.

### In vivo sub-chronic toxicity study

The main purpose of this study was to examine the effects of NLC-Eu on healthy BALB/c mice. Generally, sub-chronic toxicity study is usually conducted in one or more rodent species to determine the lethal dosage of a particular compound. For a new drug candidate, it is a requirement to determine whether the drug is toxic or not and safe for consumption by an organism. Toxicity tests are commonly done on animal models first, typically in rodents, such as rats or mice. Studies on animal models may provide essential information regarding the toxic signs and effects, as well as the recovery of the animals [49]. The clinical observation of the animals indicated that there was no obvious toxicity in NLC-Eu, NLC-Blank and eucalyptol-treated mice.

The most common and notable observations of clinical abnormality during an animal model study are the appearance of a rough coat of fur and slower or decreased activity developing during the course of this in vivo toxicity study. None of these abnormal physical characteristics were observed in all the mice groups, likewise, none of the mice showed behavioral changes or died upon 4 weeks of NLC-Eu administration. There were also no significant changes in the body weight and organ weight index between the control group and all treatment groups.

Similarly, the concentration of serum biochemical parameters also did not differ significantly among the untreated, NLC-Eu and eucalyptol treatment groups compared to the NLC-Blank. Among the parameters used to assess the toxicity effects include the biochemical analysis of treated mice serum. The reading of NLC-Eu and eucalyptol for ALP, AST, ALT, total bilirubin, creatinine and triglyceride did not exhibit toxicity reaction as seen similarly in the untreated group. Based on the serum biochemical profiles, the encapsulation of eucalyptol (NLC-Eu) remains non-toxic as there were no noteworthy disparities detected when compared to the eucalyptol treated group. Hepatic change or damage may occur upon toxicity study and it is a serious issue that should be taken into consideration when formulating a new drug or compound. Based on the results of this study, there was slight to no significant changes or toxicity damage occurred in all the treated mice. The detection of liver damage by hepatoxins or drug in the body can be indicated by the level of ALT [50]. When liver damage happen, additional AST level will be released to the blood as an indicator of a hepatic injury in the host [51]. Both ALT and AST levels correlate with one another, whereby, when the level of AST inclines, the level of ALT will also increase [52]. Elevation in the level of ALT, AST and ALP also indicate liver, gall bladder and heart complications [53]. Based on the results compared with the untreated group, the detection of slight elevations of ALT and AST levels and the disposition of fat globules as observed in the liver cross-section image of the NLC-Blank treated group may indicate the occurrence of hepatic steatosis. A previous study had characterized these microvesicular steatosis as hepatocytes which appear foamy and has nuclei located centrally [54]. Based on the liver section of NLC-Blank (Fig. 9b), despite the slight occurrence of these microvesicular vacuoles, majority of the hepatocytes in the section appear to remain compact and arranged in cords, thus, it is not significant enough to be concluded as a manifestation of liver damage. This indicates that the 28-day administration of NLC-Blank also remains non-toxic towards the mice. The elevation of creatinine level, on the other hand, indicates kidney diseases or impaired kidney function which reflects the renal functions of the host [55]. Based on the results, it can be confirmed that NLC-Eu, NLC-Blank and eucalyptol were not toxic for consumption as there were no abnormalities and no significant changes in the serum biochemical analysis parameters and creatinine level in the serum biochemical analysis.

In the assessment of NLC-Eu as a potential therapeutic compound, the histopathology images on the condition of liver and kidneys upon treatment are necessary in determining the effect of the nanoparticles on the tissues or organs of mice while observing for any abnormalities, inflammation or scarring which may occur. Histological examinations of the liver and kidneys in NLC-Eu treated group showed no abnormalities compared to the control group.

Both NO and MDA are signaling molecules that initiate inflammation under normal conditions. NO is a signaling molecule which commonly regulates immune response and apoptosis [56]. In this study, NLC-Eu had reduced the level of MDA and NO in treated mice compared to the control group (Figs. 10 and 11). Elevated level of NO is commonly regulated by cytokine-activated macrophage in the immune system and mediate metastatic process and diseases, like tumor formatting and cancers [57, 58]. Although the reduction of the NO and MDA levels were not significant, the decline indicates the potential of the formulation to exhibit anti-inflammatory attributes. In this study, it is noteworthy that the formulation did not induce any inflammation or deleterious effects towards the treated mice.

## Conclusion

NLC-Eu formulation has been successfully formulated and characterized. The NLC-Eu has a very good EE and DLC, while its PS and PDI fall into a nanometer range. NLC shows a promising potential as a good nanocarrier of poor water-soluble drug or active compound, like eucalyptol, by increasing its stability and bioavailability. It is also plausible as a cytotoxic agent as manifested by the apoptosis-inducing capability on breast cancer cells. NLC-Eu also did not cause any significant changes on the body weight and organ weight index of treated mice, as well as the serum biochemical analysis during 28 days of treatment. Altogether, NLC-Eu did not induce mortality, abnormal body weight changes and toxicological signs and effects in healthy BALB/c mice. Hence, NLC-Eu offers a safe and sustainable carrier system that has cytotoxic effect on breast cancer cell lines. Further studies need to be conducted to further study the mechanism of apoptosis induction, pharmacokinetics, pharmacodynamics and therapeutic potential of NLC-Eu in vivo to confirm its efficacy for breast cancer therapeutics.

## Data Availability

The datasets used and/or analyzed during the current study are available from the corresponding author on reasonable request.

## Abbreviations

4 T1: Mouse breast cancer cell
ACUC: Animal Care and Use Committee
ALP: Alkaline phosphatase
ALT: Alanine aminotransferase
AO: Acridine orange
AO/PI: Acridine orange and propidium iodide
AST: Aspartate aminotransferase
ATCC: American Type Cell Culture Collection
BALB/c: Mouse strain
BHT: Butylated hydroxytoluene
BW: Body weight
DLC: Drug loading capacity
DLS: Dynamic light scattering
DMEM: Dulbecco’s Modified Eagle’s Medium
DMSO: Dimethyl sulfoxide
EDTA: Ethylenediaminetetraacetic acid
EE: Entrapment efficiency
FBS: Fetal bovine serum
FDA: Food and Drug Administration
H&E: Hematoxylin and Eosin
HPO: Hydrogenated palm oil
IC: Inhibitory concentration
IC25: Inhibitory concentration at 25% from control
IC50: Inhibitory concentration at 50% from control
IL-1β: Interleukin-1β
MCF7: Human breast cancer cell
MDA: Malondialdehyde
MDA MB-231: Human breast cancer cell
MTT: 3-(4,5-dimethylthiazol-2-yl)-2,5-diphenyl tetrazolium bromide
NLC: Nanostructured Lipid Carrier
NLC-Blank: Nanostructured Lipid Carrier (treatment group)
NLC-Eu: Nanostructured Lipid Carrier loaded with Eucalyptol
NO: Nitric oxide
PDI: Polydispersity index
PI: Propidium iodide
PS: Particle size
RPMI: Roswell Park Memorial Institute Medium
SPSS: Statistical Package for the Social Sciences
T47D: Human breast cancer cell
TBA: Thiobarbituric acid
TCA: Trichloroacetic acid
TEM: Transmission Electron Microscopy
TNFα: Tumor necrosis factor-α
ZP: Zeta potential
